# Ankylosis and Its Treatment

**Published:** 1899-06-24

**Authors:** A. H. Tubby

**Affiliations:** Senior Assistant Surgeon to Westminster Hospital, Surgeon to the National Orthopædic and Evelina Hospitals


### Hospital Clinics and Medical Progress.

### ANKYLOSIS AND ITS TREATMENT.

By A. H. Tubby, M.S.Lond., F.R.C.S.Eng., Senior Assistant Surgeon to Westminster Hospital, Surgeon to the National Orthopædic and Evelina Hospitals.

Ankylosis is a permanent pathological condition of the articulations, characterised by the interposition of new tissue between the articular extremities, and diminishing or abolishing the movements natural to the part. (Rédard.) The condition known as spurious ankylosis includes that of contracture, which arises from many causes. It follows after ulceration, wounds, and burns, involving the skin and subcutaneous tissue, with retraction of the scar; after acute or chronic inflammation of fasciæ and aponeuroses, arising from gout and syphilis or rheumatism; after affections of the muscles, either rheumatic, traumatic, inflammatory, or from suppuration about and in them, and from gonorrheal arthritis, and as the result of long retention of a vicious position, as in scoliosis. Periarticular inflammations resulting from injury and rheumatism, with adhesions outside the joint and lesions of the central nervous system, as in spastic paralysis, are responsible, too, for spurious ankylosis.

To distinguish between spurious ankylosis or contracture and true ankylosis, Mr. Howard Marsh, in his work on Diseases of the Joints and Spine, says: "If care be taken, the distinction between true ankylosis and mere stiffness depending on conditions external to a joint can seldom be difficult. The patient's history is different in the two cases. In instances of ankylosis there is generally an account of either acute or prolonged inflammation of the joint itself; while if stiffness depends on conditions external to the joint, there is a history either of some accident of a slight inflammatory attack, or of merely incipient disease of the joint. If the patient be examined under an anæsthetic any stiffness that is due to muscular spasm will disappear; while, should rigidity depend on external adhesions, a very slight amount of force, tentatively applied, will often suffice to rupture some of them, and the nature of the case will become clear, not only from the fact that the adhesions as they give way are felt to be outside the joint, but from the immediate restoration of considerable movement."

In treating contractures or spurious ankylosis either gradual or operative measures may be employed. The former consist of friction, douching, and frequently-repeated passive movements. Much value has been claimed for the employment of the hot-air bath to the limb. Dry air is heated to a temperature of 250 deg. to 300 deg. Fah. in a suitable cylinder, and in this the affected limb is placed for an hour or so. Mr. Willett found that this form of treatment was useful for extra-articular adhesions, but not for cases of intra-articular fibrous ankylosis. The result of soaking contractured limbs continuously in hot water is often good, and in gouty cases it seems that if the water be made alkaline with bicarbonate of soda the parts become supple at an earlier date than if water alone is used. If such means fail, and there has not been at any time serious joint disease, forcible manipulation may be tried. When a joint has suffered severely it is very unwise to attempt to break down adhesions, as such attempts almost invariably fail; and still worse, if the disease have been tubercular in character, there is a very appreciable risk of lighting it up. Mr. Howard Marsh suggests that for forcible manipulation an anæsthetic should be employed—preferably gas. He says, "Indeed, it may be regarded as an axiom that the good to be obtained is, in the great majority of cases, inversely proportionate to the amount of force required. Only very slight force is necessary. It is well to observe that a single manipulation is frequently useless. If left to itself the joint will soon be as stiff as ever. Manipulation must be followed by hot douching and daily massage." The conditions in which manipulation is of value are, according to that surgeon: (*a*) After sprains in which extra-articular adhesions have formed, but the joint is uninjured; (*b*) after subacute rheumatism with stiffness and severe pain, in which gentle movement shows that the joint-surfaces glide smoothly on one another; (*c*) in cases in which acute or subacute rheumatism has left slight intra-articular adhesions; (*d*) in those cases where a joint becomes stiff and painful after fracture in its neighbourhood, those in which a dislocation has left a partially stiff joint, and in those stiff after a lengthened application of a splint; (*e*) after old unreduced dislocations forcible manipulation often relieves the pain, and improves the position of the limb. To the foregoing may be added (*f*) in the cases of bruised deltoid, in which any attempt to raise the arm is painful. As pointed out by Mr. Golding-Bird, the pain and stiffness are due to exudation of inflammatory material into the loose cellular tissue beneath the deltoid. Through this tissue the circumflex nerve passes, and at each movement of the deltoid the inflammatory effusion and the nerve are dragged on. It is, therefore, better to break down the adhesions.

Ankylosis in its correct surgical designation is always intra-articular. The varieties are fibrous and osseus or partial and complete. In some instances of fixed joint the fibrous and osseous forms are combined. In the centre is a variable amount of fibrous tissue, and externally is fully formed bone. For practical purposes these are cases of bony ankylosis, and no suspicion of the presence of fibrous tissue is entertained until an occasion arises for making a longitudinal section through the joint.

The immediate causes of ankylosis are all forms of acute, subacute, and chronic arthritis, and to these may be added occasional instances of Charcot's disease, osteitis deformans, and Volkmann's "cartilaginous ankylosis." Many forms of non-purulent arthritis end in osseous ankylosis, but generally it may be said that acute non-purulent forms produce fibrous ankylosis. Some cases of Pott's disease, in which no suppuration has occurred, result in bony ankylosis, and the last stages of scoliosis and osteo-arthritis of the spine are similar in their morbid anatomy. Formation of intra-articular bone is seen sometimes after gout and gonorrheal rheumatism and acute rheumatism, entirely apart from suppuration. Osteitis deformans is another case in point. Suppurative inflammation is not necessarily followed by bony ankylosis. Take, for example, many cases of suppurative tubercular arthritis of the knee-joint, in which the ankylosis is almost always fibrous. Even in some cases of prolonged suppuration in a joint recovery has followed with the preservation of considerable movement. But it will sometimes happen that in fibrous ankylosis the bands passing between the joint surfaces are so short, numerous, and strong that no movement can be obtained even under an anæsthetic.

*(To be continued.)*

